# Editorial: Cardiovascular cell senescence in aging and disease

**DOI:** 10.3389/fcvm.2023.1177395

**Published:** 2023-03-24

**Authors:** Carlo Gaetano, Maurizio Pesce, Antonio P. Beltrami, Maurizio C. Capogrossi

**Affiliations:** ^1^ Laboratorio di Epigenetica, Istituti Clinici Scientifici Maugeri IRCCS, Pavia, Italy; ^2^ Unità di Ingegneria Tissutale Cardiovascolare, Centro Cardiologico Monzino, IRCCS, Milan, Italy; ^3^ Dipartimento di Medicina, Istituto di Anatomia Patologica Universitaria, Azienda Ospedaliero Universitaria “S. Maria della Misericordia”, Udine, Italy; ^4^ Division of Cardiology, Johns Hopkins University, Baltimore, MD, United States; ^5^ Laboratory of Cardiovascular Science, National Institute on Aging, National Institutes of Health, Baltimore, MD, United States

**Keywords:** aging, senescence, signal transduction, transcriptomic, cardiovascular disease

**Editorial on the Research Topic** Cardiovascular cell senescence in aging and disease

There is no uniform consensus on the relationships between age and aging. For example, the Medical Subject Heading definition in Medline defines aging as “The gradual, irreversible changes in structure and function of an organism that occur as a result of the passage of time.” However, such a definition has severe flaws because it fails to include the broader context of development in early life, plasticity, and regeneration. For example, in the cardiovascular system, the plasticity of the blood vessels, including their ability to regenerate in response to a variety of cues, is in contrast to the minimal regenerative ability of the myocardium; and cardiovascular coupling increases the complexity of such an articulated organismal system in response to aging. In this context, the impact of the aging process and the associated cellular senescence makes it challenging to predict the impact of aging on the different cardiovascular components (1). Moreover, the concept that aging related to cellular senescence is only a function of time is misleading, at least for the cardiovascular system. Consolidated evidence suggests that the combination of risk conditions can have a variable impact on cellular senescence, translating into accelerated biological aging.

It is noteworthy that the risk of developing cardiovascular disease (CVD) is primarily (75%–90%) explained by the presence or absence of traditional CVD risk factors (2). An increase in age is a well-known traditional risk factor generally considered non-modifiable. In 2021, the American Heart Association (AHA) reported that the incidence of CVD in US men and women is ∼40% from 40 to 59 years; the incidence rises in the 60–79-year-old age group to 77.5% of males and 75.4% of females; in the 80 plus;-year-old age group, CVD incidence reaches 89.4% in males and 90.8% in females (https://professional.heart.org/-/media/PHD-Files-2/Science-News/2/2021-Heart-and-Stroke- StatUpdate/2021_Stat_Update_factsheet _Older _and_CVD.pdf.).

The burden of CVD is directly related to the increased mortality, morbidity, and frailty in affected individuals, which also translates to significant overall healthcare costs (3). According to the World Population Prospects 2022 (https://population.un.org/wpp/), the share of the global population aged 65 years or above is projected to rise from 10 percent in 2022 to 16 percent in 2050. By then, 1 in 6 people worldwide will be over 65 with a clear potential of developing cardiovascular accidents just because of age.

In this scenario, investigating the molecular mechanisms associated with cardiovascular aging and its pathophysiological consequences is of the utmost relevance. The special issue of Frontiers in Cardiovascular Medicine dedicated to “Cardiovascular Cell Senescence in Aging and Disease” goes well in this direction.

In a first experimental article, Wang et al. experimentally explored the molecular role of the poorly characterized E3 ubiquitin ligase named mitsugumin 53 (MG53) in regulating necroptosis following ischemia- reperfusion injury (I/R) injury to the heart and the involvement of reactive oxygen species (ROS) in MG53-mediated cardioprotection. The authors conclude that appropriate modulation of ROS is crucial in limiting the consequences of ischemic cardiac damage.

In another study, Gou et al. applied single-cell transcriptomics to understand how cellular senescence might negatively impact the mouse vascular endothelium. They found that ribosome biogenesis, inflammation, apoptosis, and angiogenesis-related genes and pathways changed with age and that the transcription factor *Jun* could be implicated in this process.

In the same issue, a review from Liu et al. addresses the anti- aging properties of physical exercise. Here, the authors claim that there is substantial evidence that changes in the autonomic nervous system and arterial stiffness play an essential role in the development of cardiovascular disease during the aging process. In this context, exercise is known to be effective in improving autonomic regulation and arterial vascular compliance, but differences in the type and intensity of exercise can have varying degrees of impact on vascular regulatory responses and autonomic function.

Finally, in a valuable contribution from Dominga Iacobazzi of Paolo Madeddu's group (Iacobazzi et al.), it is proposed that patients with congenital heart disease (CHD suffer from multiple repeated stresses from an early stage life, which wears out homeostatic mechanisms and cause premature cardiac aging.

In conclusion, consolidating cell senescence and aging as essential effectors of cardiovascular risk, this special issue of Frontiers in Cardiovascular Medicine emphasizes still underestimated aspects. The role of ubiquitination, transcription factors, exercise, and congenital heart disease are only apparently heterogeneous. They are different aspects of the same problem, namely, the effect of aging progression on the cardiovascular system function. Potential and novel strategies to reduce the negative impact of aging as a risk factor for heart disease may arise from reading these contributions.
